# Supplementary material for: Small variant benchmark from a complete assembly of X and Y chromosomes
Source: Nat Commun. 2025 Jan 8;16:497. doi: 10.1038/s41467-024-55710-z (PMC11711550; doi:10.1038/s41467-024-55710-z)
Supplement: Supplementary file 1 — Supplementary Information [file 41467_2024_55710_MOESM1_ESM.pdf]

Supplementary Information for:

Small variant benchmark from a complete assembly of X and Y chromosomes

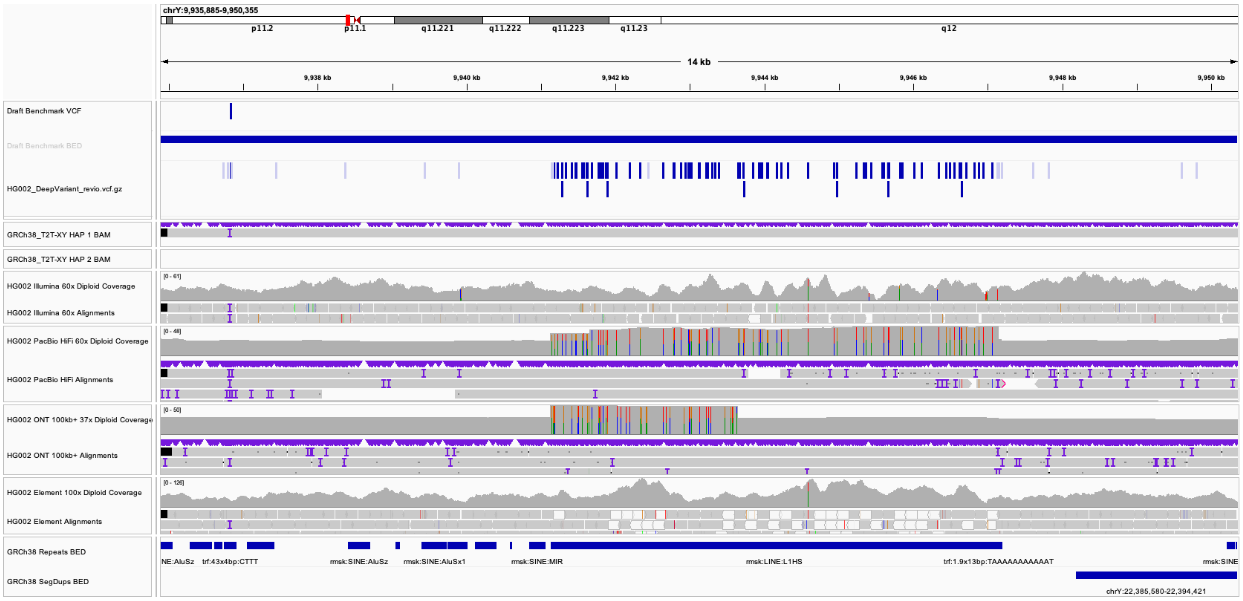

Supplementary Figure 1:.Region with many false positives in mapping-based methods. DeepVariant PacBio Revio HiFi calls here have many false positives due to additional divergent copies of sequence in HG002 not in GRCh38. The assembly resolves the correct sequence in this region (chrY:9,941,135-9,947,154), with only one variant relative to GRCh38. Mapping-based approaches can result in many false positives due to differences in reads mis-mapping from the additional copies of this sequence in HG002.

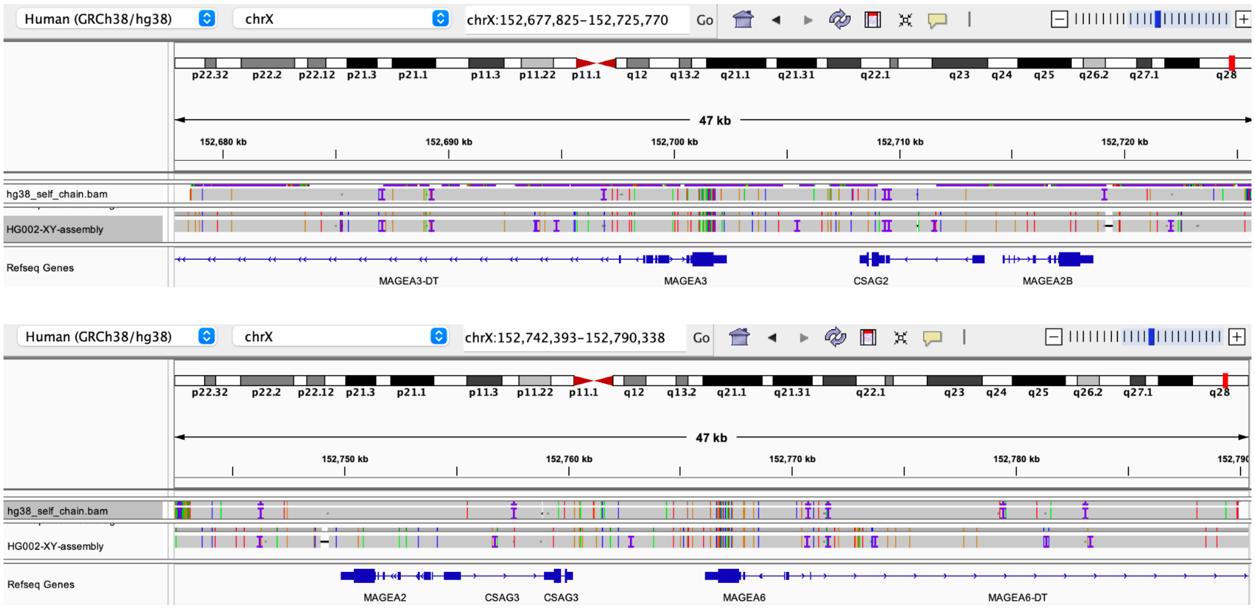

Supplementary Figure 2. Example Gene Conversion. IGV visualization showing that many of the variants in *MAGEA3* and *CSAG2* (top panel), and *MAGEA6* (bottom panel) in HG002 XY assembly match the variants in the self-chain alignment of the segmental duplication, indicating a gene conversion-like event.

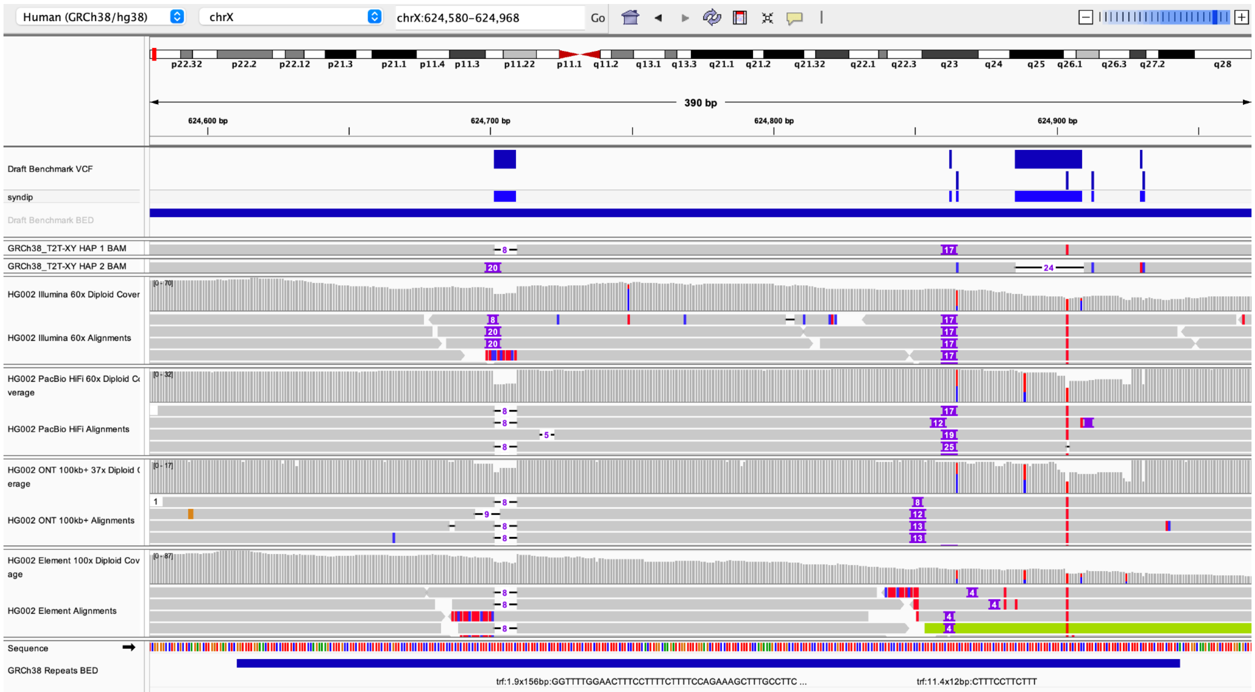

Supplementary Figure 3:.Complex variants in a long tandem repeat in the PAR. In this region, the assemblies used for the benchmark accurately resolve horizontally complex variants, with multiple variants in each haplotype, resulting in phased variant calls that can be used for benchmarking.
