# Supplementary material for: Small variant benchmark from a complete assembly of X and Y chromosomes
Source: Nat Commun. 2025 Jan 8;16:497. doi: 10.1038/s41467-024-55710-z (PMC11711550; doi:10.1038/s41467-024-55710-z)
Supplement: Supplementary file 2 — Description of Additional Supplementary Files [file 41467_2024_55710_MOESM2_ESM.pdf]

**Description of Additional Supplementary Files**

File Name: Supplementary  Data 1
Description: Detailed results from Manual Curation of Benchmark. External curations are in columns B-D, and NIST recurated any variants that may be incorrect in the benchmark in columns E-I. Curations in bold differed from initial curation and were returned to the initial curators to get consensus. Remaining columns contain annotations of repeats from active evaluation

File Name: Supplementary Data 2
Description: Active Evaluation results. We calculated 95% confidence intervals for whether the benchmark reliably identifies errors in each stratum. The column sys_conf_lower gives the lower bound of the confidence intervals.

File Name: Supplementary Data 3
Description: Long Range PCR and Sanger Validation for Subset of Variants. Includes numbers of variants confirmed and contradicted in each gene, primer sequences, Long Range PCR Reaction Components and PCR conditions.

File Name: Supplementary Data 4
Description: Stratified benchmarking results from hap.py extended.csv for HiFi-DeepVariant calls from 2020 against GIABv4.2.1 benchmark.

File Name: Supplementary  Data 5
Description: Stratified benchmarking results from hap.py extended.csv for HiFiDeepVariants calls from 2020 against GIAB Challenging Medically Relevant Genes benchmark.

File Name: Supplementary Data 6
Description: Stratified benchmarking results from hap.py extended.csv for HiFiDeepVariants calls from 2020 against GIAB XY v1.0 benchmark.

File Name: Supplementary Data 7
Description: Stratified benchmarking results from hap.py extended.csv for HiFi-DeepVariant calls from 2023 against GIABv4.2.1 benchmark.

File Name: Supplementary Data 8
Description: Stratified benchmarking results from hap.py extended.csv for HiFiDeepVariants calls from 2023 against GIAB Challenging Medically Relevant Genes benchmark.

File Name: Supplementary Data 9
Description: Stratified benchmarking results from hap.py extended.csv for HiFiDeepVariants calls from 2023 against GIAB XY v1.0 benchmark.
