# Supplementary material for: Small variant benchmark from a complete assembly of X and Y chromosomes
Source: Nat Commun. 2025 Jan 8;16:497. doi: 10.1038/s41467-024-55710-z (PMC11711550; doi:10.1038/s41467-024-55710-z)
Supplement: Supplementary file 12 — Reporting Summary [file 41467_2024_55710_MOESM12_ESM.pdf]

nature portfolio

Corresponding author(s): Justin M. Zook

Last updated by author(s): 11/10/2024

Reporting Summary

Nature Portfolio wishes to improve the reproducibility of the work that we publish. This form provides structure for consistency and transparency in reporting. For further information on Nature Portfolio policies, see our Editorial Policies and the Editorial Policy Checklist.

Statistics

For all statistical analyses, confirm that the following items are present in the figure legend, table legend, main text, or Methods section.

n/a	Confirmed	
☐	☒	The exact sample size (*n*) for each experimental group/condition, given as a discrete number and unit of measurement
☐	☒	A statement on whether measurements were taken from distinct samples or whether the same sample was measured repeatedly
☒	☐	The statistical test(s) used AND whether they are one- or two-sided<br>*Only common tests should be described solely by name; describe more complex techniques in the Methods section.*
☒	☐	A description of all covariates tested
☒	☐	A description of any assumptions or corrections, such as tests of normality and adjustment for multiple comparisons
☒	☐	A full description of the statistical parameters including central tendency (e.g. means) or other basic estimates (e.g. regression coefficient) AND variation (e.g. standard deviation) or associated estimates of uncertainty (e.g. confidence intervals)
☒	☐	For null hypothesis testing, the test statistic (e.g. *F*, *t*, *r*) with confidence intervals, effect sizes, degrees of freedom and *P* value noted<br>*Give P values as exact values whenever suitable.*
☒	☐	For Bayesian analysis, information on the choice of priors and Markov chain Monte Carlo settings
☒	☐	For hierarchical and complex designs, identification of the appropriate level for tests and full reporting of outcomes
☒	☐	Estimates of effect sizes (e.g. Cohen's *d*, Pearson's *r*), indicating how they were calculated

Our web collection on statistics for biologists contains articles on many of the points above.

Software and code

Policy information about availability of computer code

Data collection	No software used
Data analysis	The code used to generate the XY benchmark is at https://github.com/nate-d-olson/defrabb/tree/b0f08b6b0514555570e8f90fa51b0a86a3c904da. The config files used for the defrabb run, include input files for the repeats, are at https://github.com/nate-d-olson/defrabb/blob/b0f08b6b0514555570e8f90fa51b0a86a3c904da/config/analyses_20230315_v0.011-HG002XY.tsv and https://github.com/nate-d-olson/defrabb/blob/b0f08b6b0514555570e8f90fa51b0a86a3c904da/config/resources.yml. This pipeline include dipcall v0.3 and minimap2 (v2.24). We refined the benchmark using an active evaluation approach available at https://github.com/usnistgov/active-evaluation.<br>The code to create the exclusion bed files is at https://github.com/jmcdani/giab-chrXY-benchmark/blob/main/scripts/chrXY_benchmark_exclusions.ipynb

For manuscripts utilizing custom algorithms or software that are central to the research but not yet described in published literature, software must be made available to editors and reviewers. We strongly encourage code deposition in a community repository (e.g. GitHub). See the Nature Portfolio guidelines for submitting code & software for further information.

Data

Policy information about availability of data

All manuscripts must include a data availability statement. This statement should provide the following information, where applicable:

- Accession codes, unique identifiers, or web links for publicly available datasets
- A description of any restrictions on data availability
- For clinical datasets or third party data, please ensure that the statement adheres to our policy

The benchmark vcf and bed, as well as supporting files, are available at https://ftp.ncbi.nlm.nih.gov/ReferenceSamples/giab/release/AshkenazimTrio/HG002_NA24385_son/chrXY_v1.0/. The sequencing data used in this study are available in the NCBI SRA database under accession code PRJNA200694 [http://www.ncbi.nlm.nih.gov/bioproject/PRJNA200694].

Research involving human participants, their data, or biological material

Policy information about studies with human participants or human data. See also policy information about sex, gender (identity/presentation), and sexual orientation and race, ethnicity and racism.

Reporting on sex and gender	To include both chromosomes X and Y, we focus analysis on one individual of male sex with extensive sequencing data and complete assembly of these chromosomes.
Reporting on race, ethnicity, or other socially relevant groupings	This individual self-reported to be of Ashkenazi Jewish ancestry to the Harvard Personal Genome Project
Population characteristics	This individual self-reported to be of Ashkenazi Jewish ancestry to the Harvard Personal Genome Project
Recruitment	No recruitment performed in this study.
Ethics oversight	This research complies with all relevant ethical regulations, and was approved by the NIST Research Protections Office. This individual (male, with X and Y chromosomes) was consented under the Harvard Personal Genome Project.(Ball et al. 2012)

Note that full information on the approval of the study protocol must also be provided in the manuscript.

Field-specific reporting

Please select the one below that is the best fit for your research. If you are not sure, read the appropriate sections before making your selection.

☒ Life sciences ☐ Behavioural & social sciences ☐ Ecological, evolutionary & environmental sciences

For a reference copy of the document with all sections, see nature.com/documents/nr-reporting-summary-flat.pdf

Life sciences study design

All studies must disclose on these points even when the disclosure is negative.

Sample size	Only one sample is used because it was the only sample with complete and polished X and Y assemblies
Data exclusions	No data were excluded
Replication	Multiple sequencing technologies were used to measure this sample and evaluate the benchmark
Randomization	Not relevant because only one sample was used
Blinding	No blinding because only one sample was used and all data was previously public

Reporting for specific materials, systems and methods

We require information from authors about some types of materials, experimental systems and methods used in many studies. Here, indicate whether each material, system or method listed is relevant to your study. If you are not sure if a list item applies to your research, read the appropriate section before selecting a response.

Materials & experimental systems

n/a	Involved in the study	
☒	☐	Antibodies
☐	☒	Eukaryotic cell lines
☒	☐	Palaeontology and archaeology
☒	☐	Animals and other organisms
☒	☐	Clinical data
☒	☐	Dual use research of concern
☒	☐	Plants

Methods

n/a	Involved in the study	
☒	☐	ChIP-seq
☒	☐	Flow cytometry
☒	☐	MRI-based neuroimaging

Eukaryotic cell lines

Policy information about cell lines and Sex and Gender in Research

Cell line source(s)	DNA is extracted from publicly available cell lines GM24385 (HG002, RRID:CVCL_1C78) at the Coriell Institute for Medical Research National Institute for General Medical Sciences cell line repository.
Authentication	Authenticated by examining concordance of variants with previous studies
Mycoplasma contamination	Cell line tested negative for mycoplasma contamination.
Commonly misidentified lines (See ICLAC register)	None

Plants

Seed stocks	N/A
Novel plant genotypes	N/A
Authentication	N/A
